# Efficient Photocatalytic Elimination of Imidazolinone Herbicides by Bismuth-Based Photocatalyst BiOIO_3_

**DOI:** 10.3390/molecules31081361

**Published:** 2026-04-21

**Authors:** Weili Yu, Yan Tian, Mengyu Guo, Shuping Tong, Chengshuai Li, Bingjie Zhang, Yongqiang Ma

**Affiliations:** 1 Shandong Key Laboratory for Green Prevention and Control of Agricultural Pests, Shandong Academy of Pesticide Sciences, Jinan 250033, China; 313304200@163.com (W.Y.); mengyuguo566@163.com (M.G.); 13989046652@163.com (S.T.); liucling2004@163.com (C.L.); 2 Guangxi Zhuang Autonomous Region Ecological Environment Monitoring Center, Nanning 530012, China; ttyezi96@163.com; 3 Department of Applied Chemistry, College of Science, China Agricultural University, Beijing 100193, China; mayongqiang@cau.edu.cn

**Keywords:** BiOIO_3_, photocatalytic, imidazolinone herbicides, high-efficiency, internal polarized electric field

## Abstract

Imidazolinone herbicides such as imazethapyr (IMT) pose potential ecological risks due to their high mobility and ecotoxicity. This study synthesized the bismuth-based photocatalyst BiOIO_3_ via a facile hydrothermal method and systematically characterized its physicochemical properties. BiOIO_3_ features a 2D lamellar structure, pure phase composition, and a built-in internal polarization electric field that efficiently separates photogenerated electron–hole pairs. Photocatalytic experiments exhibited that BiOIO_3_ achieved 84.5% elimination of IMT, with a rate constant 66 times higher than that of TiO_2_ (Rutile). Mechanistic studies revealed that photogenerated electrons (e^−^), holes (h^+^), and superoxide radicals (·O_2_^−^) are the primary reactive species. HPLC-MS/MS identified key intermediates, and QSAR-based toxicity prediction showed reduced mutagenicity for most intermediates. Importantly, BiOIO_3_ effectively eliminated five imidazolinone herbicides simultaneously. This work highlights BiOIO_3_ as a promising photocatalyst for efficient and practical remediation of imidazolinone herbicide-contaminated water.

## 1. Introduction

Imidazolinone herbicides, first developed by the American Cyanamid Company in the early 1980s, constitute a class of highly active, broad-spectrum herbicides with the advantage of controlling both monocotyledonous and dicotyledonous weeds [1]. Due to their low application rates and broad crop selectivity, imidazolinone herbicides have been widely used in agricultural production, particularly for effective control of both broadleaf and grass weeds in soybean fields [2]. Imidazolinone herbicides are extensively applied across North America, South America, and the Asia-Pacific region, with major consuming countries including the United States, Canada, Brazil, Argentina, China and India. Nevertheless, the use of imidazolinone herbicides is restricted within the European Union, where only a limited number of varieties are authorized. Imazamox (IMM) has gained rapid popularity in sunflower production across Central and Eastern Europe in recent years [3]. A number of studies have documented the occurrence of imidazolinone herbicides in agricultural soils. When applied at recommended rates, their concentrations in soil generally fall within the range of 0.01–2 mg/kg during the crop-growing period [4,5].

Among them, imazethapyr (IMT), a representative herbicide belonging to the imidazolinone family, is characterized by low soil adsorption capacity, which renders it susceptible to leaching and runoff into aquatic ecosystems [6,7]. Such mobility inevitably leads to adverse impacts on the ecological environment. Previous studies have demonstrated that IMT exerts specific toxic effects on the rainbow trout gill cell line (RTgill-W1), highlighting its potential ecotoxicological risks [8]. Therefore, the environmental risks associated with IMT cannot be overlooked and warrant extensive attention from the research community. It is imperative to develop efficient technologies and methods for the elimination of imazethapyr and other imidazolinone herbicides from contaminated water environments.

At present, the main remediation technologies for organic pollutants include biological, physical and chemical methods [9]. Biological methods are mainly realized through the metabolic activities of microorganisms and possess the advantages of sustainability and environmental friendliness. However, the screening and cultivation of microorganisms are relatively difficult, and they are susceptible to environmental factors [10]. Among physical methods, adsorption, the most widely applied technique, is competitive due to its simple operation, high efficiency and low cost [11]. Nevertheless, physical adsorption essentially achieves the transfer of pollutants rather than their complete elimination. Chemical oxidation decomposes pollutants using oxidants such as chlorine gas, sodium hypochlorite, and potassium permanganate. But the oxidants themselves exert negative impacts on environmental organisms, thus restricting their application and popularization in environmental pollution remediation [12].

Advanced oxidation processes (AOPs) mainly include Fenton processes, electrochemical oxidation, photocatalytic processes, etc. Among the diverse strategies for the elimination of organic pollutants, photocatalysis stands out as an environmentally benign technology owing to its robust redox capability and ability to utilize solar energy, a renewable resource [13,14,15]. Nevertheless, the widespread application of photocatalysis in environmental remediation is severely hindered by the rapid recombination of photogenerated electron–hole pairs, which drastically diminishes its quantum efficiency [16,17,18]. Despite this bottleneck, photocatalysis remains one of the most ideal and promising approaches for pollutant degradation [19,20]. In a photocatalytic reaction system, the rational design and selection of appropriate semiconductor photocatalysts are of paramount importance.

**Figure 1:**
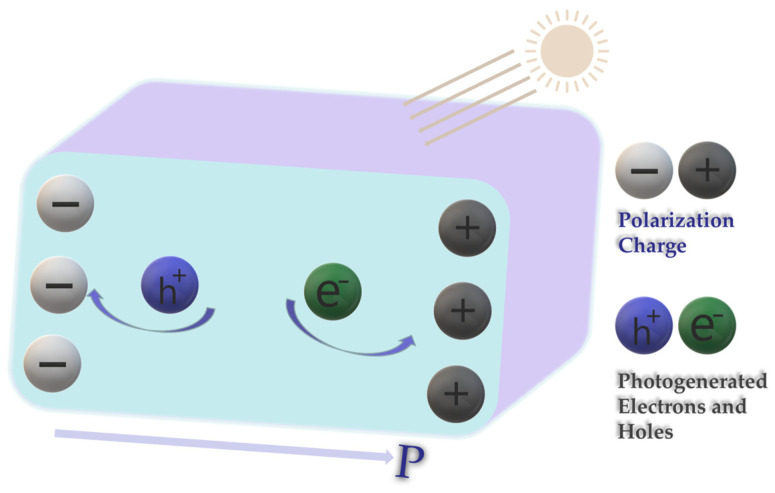
Schematic diagram of polarized electric field promoting photogenerated electron–hole separation.

BiOIO_3_ has emerged as a crystalline semiconductor material in recent years, exhibiting exceptional photocatalytic properties [21,22,23]. It possesses a favorable band structure and a built-in macroscopic internal polarization electric field [24,25]. As illustrated in Figure 1, the non-centrosymmetric configuration of ions or ionic groups within its crystal lattice results in the misalignment of the centers of positive and negative charges. This unique structural characteristic induces the formation of positively charged (C+ region) and negatively charged (C− region) domains on the material surface, generating an internal polarization electric field directed from the C− region to the C+ region (P). This internal polarization electric field serves as a driving force to effectively separate the photogenerated charge carriers, thereby significantly enhancing the overall photocatalytic performance [26,27,28].

In this study, we successfully fabricated the semiconductor photocatalyst BiOIO_3_ via a facile hydrothermal method. The as-obtained BiOIO_3_ was employed for the photocatalytic elimination of IMT and four other representative imidazolinone herbicides. A series of systematic characterizations were first conducted to comprehensively investigate the physicochemical properties of the obtained BiOIO_3_, including its micro-morphology, chemical composition and crystal structure. Subsequently, the photocatalytic performance of BiOIO_3_ for the elimination of IMT was systematically evaluated and compared with that of conventional benchmark photocatalysts, such as titanium dioxide (TiO_2_) and tungsten trioxide (WO_3_). Furthermore, in-depth investigations were carried out to elucidate the underlying photocatalytic mechanism of BiOIO_3_ towards the elimination of IMT. The intermediate products generated during the photocatalytic elimination of IMT were identified and analyzed, and the potential developmental toxicity and mutagenicity of these intermediates was predicted. Finally, the practical applicability of the as-obtained BiOIO_3_ was validated by realizing the simultaneous photocatalytic elimination of the five selected imidazolinone herbicides.

## 2. Results and Discussion

### 2.1. Characterization

#### 2.1.1. Morphological Analysis of BiOIO_3_

**Figure 2:**
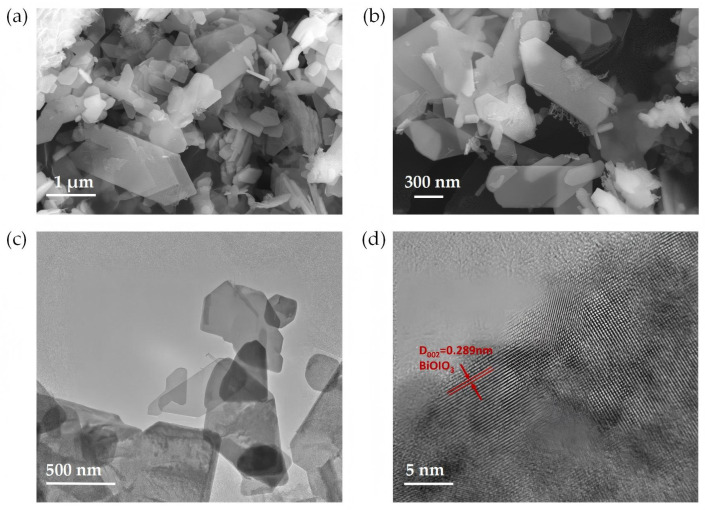
(**a**,**b**) SEM images of as-obtained BiOIO_3_; (**c**) TEM images of as-obtained BiOIO_3_; (**d**) HRTEM images of as-obtained BiOIO_3_.

The morphological characteristics of the as-obtained BiOIO_3_ were investigated by Scanning Electron Microscopy (SEM) and Transmission Electron Microscopy (TEM), as illustrated in Figure 2a–c. The SEM and TEM images clearly reveal that the BiOIO_3_ sample exhibits a typical 2D lamellar stacking structure, which is consistent with previously reported observations [29,30]. These nanosheets exhibit a relatively wide size distribution ranging from approximately 300 to 1000 nm, while maintaining a uniform and smooth surface morphology. Such a two-dimensional nanostructure is favorable for enhancing surface area and exposing more active sites. Further structural analysis was performed via High-Resolution Transmission Electron Microscopy (HRTEM), as shown in Figure 2d. A distinct lattice fringe with an interplanar spacing of 0.289 nm is clearly resolved, corresponding to the (002) crystal plane of BiOIO_3_ [31]. This observation not only confirms the successful formation of the BiOIO_3_ phase but also indicates the high crystallinity of the prepared nanosheets.

#### 2.1.2. Specific Surface Area and Pore Size Analysis

**Figure 3:**
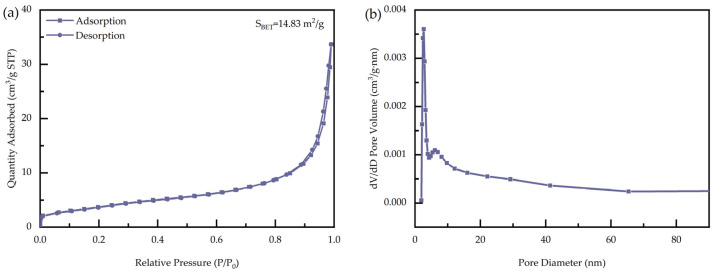
(**a**) Nitrogen adsorption–desorption isotherms of as-obtained BiOIO_3_; (**b**) pore diameter distribution of as-obtained BiOIO_3_.

Nitrogen adsorption–desorption measurements were carried out to investigate the specific surface area and pore size distribution of the as-prepared BiOIO_3_ sample. As depicted in Figure 3a, the obtained nitrogen adsorption–desorption isotherm is a Type IV isotherm with an H3 hysteresis loop, confirming the existence of a mesoporous structure in BiOIO_3_ [32,33]. The specific surface area of BiOIO_3_ was calculated to be 14.83 m^2^/g, indicating a relatively low surface area for this material. This result also indirectly reflects that the excellent photocatalytic performance of BiOIO_3_ for the elimination of IMT is largely attributed to the internal polarization electric field, which effectively enhances the separation efficiency of photogenerated electron–holes. Furthermore, it could also be due to a suitable band structure and favorable surface microenvironment. Furthermore, the pore size distribution analysis reveals that the pore diameters of BiOIO_3_ are primarily concentrated in two distinct ranges: the micropore-to-mesopore range of 1–3 nm and the wider mesopore range of 5–60 nm.

#### 2.1.3. X-Ray Powder Diffraction (XRD) Patterns Analysis

**Figure 4:**
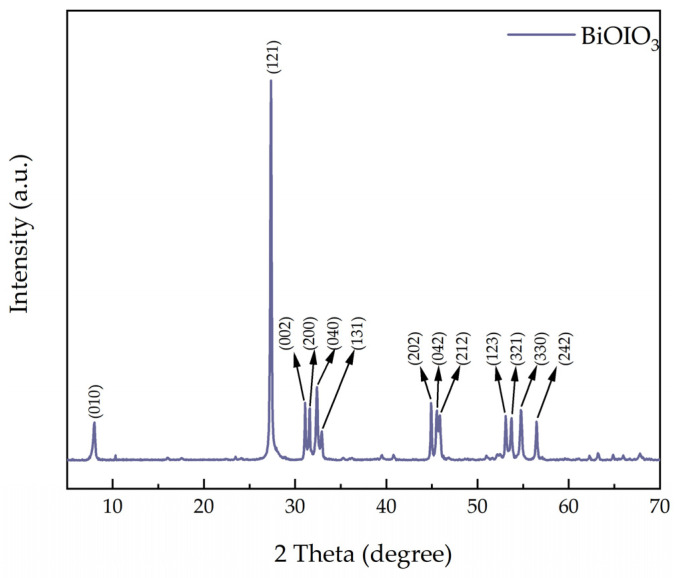
XRD pattern of as-obtained BiOIO_3_.

The XRD pattern of BiOIO_3_ is presented in Figure 4. It can be clearly observed that all of the diffraction peaks of the as-obtained BiOIO_3_ sample are in good agreement with the standard data from the JCPDS card, No. 26-2019 [34]. From left to right, these distinct diffraction peaks are sequentially assigned to the (010), (121), (002), (200), (040), (131), (202), (042), (212), (123), (321), (330), and (242) crystal planes of BiOIO_3_, respectively. The excellent match between the experimental pattern and the standard reference data confirms the successful synthesis of pure-phase BiOIO_3_ without any detectable impurity phases [35].

#### 2.1.4. Fourier Transform Infrared (FTIR) Spectra Analysis

**Figure 5:**
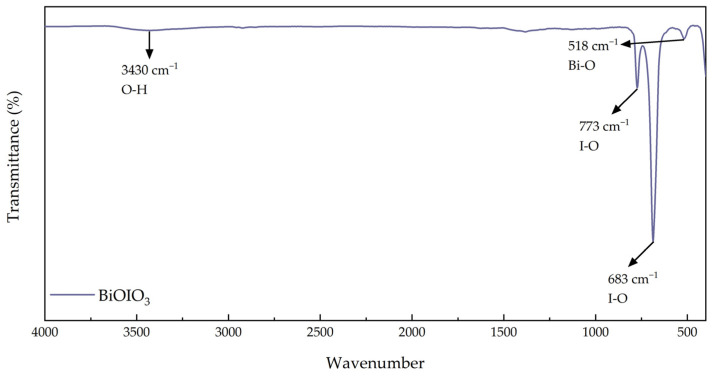
FTIR spectra of as-obtained BiOIO_3_.

The as-obtained BiOIO_3_ sample was characterized by FTIR spectra, and the corresponding spectrum is displayed in Figure 5. The characteristic absorption peaks located at 683 cm^−1^ and 773 cm^−1^ are assigned to the stretching vibrations of the I-O bonds in BiOIO_3_. The distinct vibrational peak at 518 cm^−1^ is attributed to the Bi-O bond vibrations of the material. Additionally, the broad absorption band centered at 3430 cm^−1^ corresponds to the O-H stretching vibrations originating from the adsorbed water molecules on the sample surface [36,37,38,39].

#### 2.1.5. X-Ray Photoelectron Spectroscopy (XPS) Analysis

**Figure 6:**
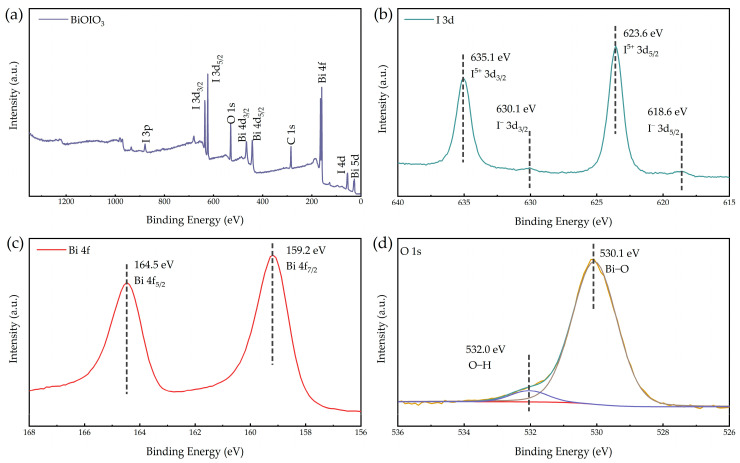
(**a**) Survey XPS spectra, (**b**) I 3d spectra, (**c**) Bi 4f spectra, and (**d**) O 1s spectra of as-obtained BiOIO_3_.

To further characterize the chemical valence states of the constituent elements in BiOIO_3_, XPS analysis was carried out, and the corresponding results are presented in Figure 6a–d. As shown in the XPS survey spectrum of BiOIO_3_ (Figure 6a), the characteristic signals of iodine (I), oxygen (O), and bismuth (Bi) elements can be clearly detected, confirming the elemental composition of the as-obtained sample [40].

The high-resolution XPS spectra of individual elements were analyzed to determine their chemical states. As depicted in Figure 6b, the peaks located at 623.6 eV and 635.1 eV are assigned to I^5+^ 3d_5/2_ and I^5+^ 3d_3/2_, respectively, while the peaks at 618.6 eV and 630.1 eV correspond to I^−^ 3d_5/2_ and I^−^ 3d_3/2_ [41]. In the high-resolution Bi 4f spectrum (Figure 6c), two distinct peaks centered at 159.2 eV and 164.5 eV are attributed to Bi 4f_7/2_ and Bi 4f_5/2_, respectively [42]. For the O 1s spectrum (Figure 6d), the peak at 530.1 eV is associated with the Bi-O bonds in the BiOIO_3_ lattice [43,44] and the peak at 531.9 eV is ascribed to the O-H bonds originating from adsorbed water molecules on the sample surface [45].

#### 2.1.6. Optical and Electrochemistry Properties

**Figure 7:**
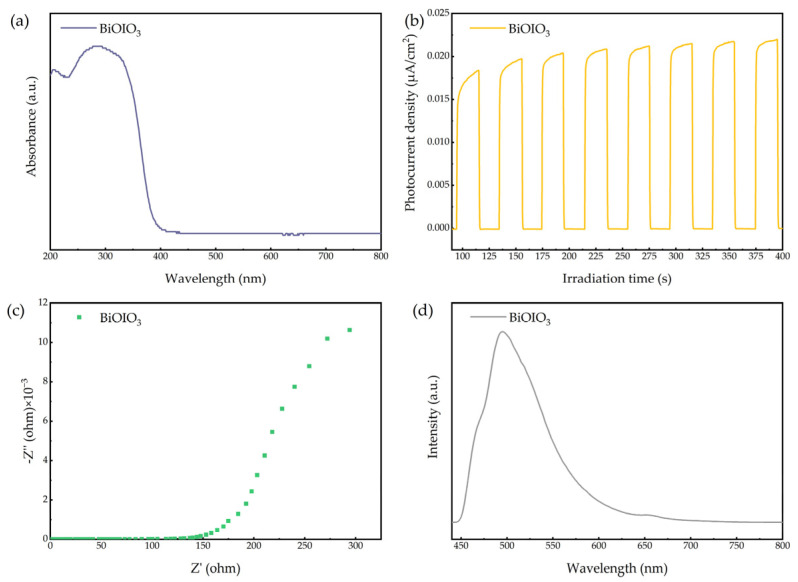
UV–Vis DRS spectra (**a**), TPC responses (**b**), EIS Nyquist plots (**c**), and PL spectra (**d**) (excitation wavelength of 420 nm) of as-obtained BiOIO_3_.

The optical properties of the as-obtained BiOIO_3_ were investigated by UV–Vis diffuse reflectance spectroscopy (UV–Vis DRS). As depicted in Figure 7a, BiOIO_3_ demonstrates ultraviolet light absorption capabilities. The photocatalytic activity of the material is predominantly governed by the separation and migration efficiency of charge carriers. For this reason, their separation and transfer behaviors were evaluated by transient photocurrent (TPC) measurements, electrochemical impedance spectroscopy (EIS) and steady-state photoluminescence (PL) spectra. As shown in Figure 7b, the TPC response of BiOIO_3_ is about 0.02 μA/cm^2^. Figure 7c,d display the EIS and PL results.

### 2.2. Photocatalytic Elimination of IMT

**Figure 8:**
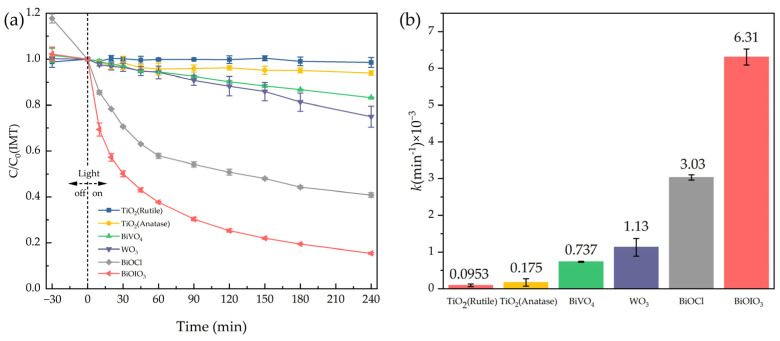
(**a**) Photocatalytic elimination of IMT and (**b**) photocatalytic elimination rat constant (*k*) of commercial TiO_2_ (Rutile and Anatase), BiVO_4_, WO_3_, BiOCl and as-obtained BiOIO_3_.

The photocatalytic elimination curve of IMT by the as-obtained BiOIO_3_ is depicted in Figure 8a, with the photocatalytic performance of BiOIO_3_ further compared with three conventional and widely investigated photocatalysts (namely TiO_2_—Rutile, TiO_2_—Anatase, BiVO_4_, WO_3_ and BiOCl). The obtained results demonstrated that BiOIO_3_ exhibited outstanding photocatalytic elimination activity toward IMT: a remarkable elimination efficiency of 84.5% for IMT was achieved after 240 min of light irradiation. In sharp contrast, the elimination efficiencies of IMT over TiO_2_—Rutile, TiO_2_—Anatase, BiVO_4_, WO_3_ and BiOCl under identical experimental conditions were merely 1.4%, 6.1%, 16.8%, 25.1% and 59.1%, respectively, which were drastically lower than that over BiOIO_3_.

To quantitatively evaluate and compare the photocatalytic elimination of IMT over the as-tested photocatalysts, the experimental elimination curves were further fitted with the pseudo-first-order kinetic model expressed as ln(C/C_0_) = −*k*t (where C represents the residual concentration of IMT at irradiation time t, C_0_ is the initial concentration of IMT, *k* is the pseudo-first-order rate constant, and t is the light irradiation time) [46]. The corresponding pseudo-first-order rate constants derived from the kinetic fitting are presented in Figure 8b. Notably, BiOIO_3_ possessed the highest elimination rate constant for IMT (0.00631 min^−1^) among all the tested photocatalysts, which was approximately 66 times higher than that of TiO_2_—Rutile, manifesting the ultra-fast photocatalytic elimination kinetics of BiOIO_3_ toward IMT.

### 2.3. Research on the Mechanism of Photocatalytic Elimination IMT by BiOIO_3_

#### 2.3.1. Reactive Species Trapping Experiments

**Figure 9:**
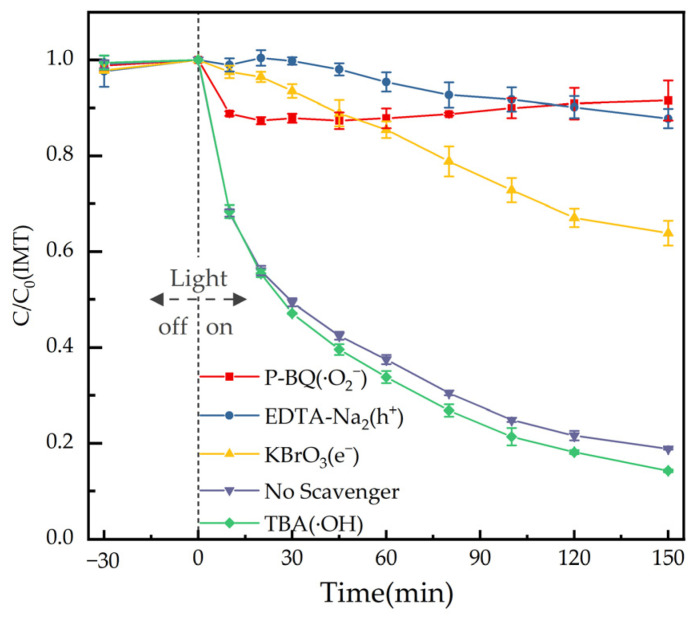
Reactive species trapping experiments of as-obtained BiOIO_3_ for photocatalytic elimination of IMT.

To identify the dominant reactive oxygen species (ROS) involved in the photocatalytic elimination of IMT over the BiOIO_3_ photocatalyst, a series of radical trapping experiments were systematically conducted under the same photocatalytic reaction conditions. Potassium bromate (KBrO_3_), tert-butanol (TBA), disodium ethylenediaminetetraacetate (EDTA-Na_2_), and p-benzoquinone (p-BQ) were selected as the specific scavengers, which were employed to capture photogenerated electrons (e^−^), hydroxyl radicals (·OH), photogenerated holes (h^+^), and superoxide radicals (·O_2_^−^), respectively. The photocatalytic elimination curves of IMT in the presence of different scavengers are presented in Figure 9. The experimental results revealed that the introduction of TBA exerted a slight promotional effect on the photocatalytic elimination reaction of IMT, implying that ·OH was not the active species responsible for the elimination process. In stark contrast, the addition of KBrO_3_, EDTA-Na_2_ and p-BQ led to a significant and obvious inhibition of the photocatalytic elimination IMT by BiOIO_3_. These results clearly demonstrated that e^−^, h^+^ and ·O_2_^−^ served as the primary ROS.

#### 2.3.2. Analysis of the Band Position of BiOIO_3_

**Figure 10:**
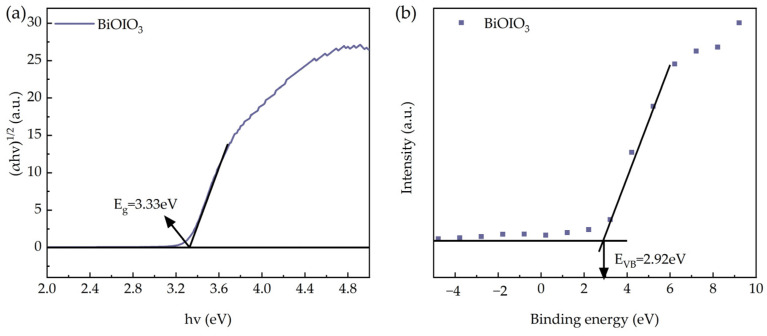
(**a**) Band gaps and (**b**) XPS valence band of as-obtained BiOIO_3_.

The UV–Vis DRS spectrum of the as-obtained BiOIO_3_ is presented in Figure 7a. BiOIO_3_ exhibits a predominant light absorption range in the ultraviolet region with almost no absorption in the visible region. Based on the Tauc/Davis–Mott model (αhv)^1/2^ = A(hv − E_g_) and the UV–Vis DRS data, the band gap (E_g_) of BiOIO_3_ was calculated and the results are shown in Figure 10a. The E_g_ value of BiOIO_3_ is determined to be 3.33 eV. XPS valence band spectroscopy was employed to characterize the valence band (E_VB_) position, with the E_VB_ of BiOIO_3_ being 2.92 eV as displayed in Figure 10b. The conduction band (E_CB_) position of BiOIO_3_ was calculated to be −0.41 eV using the formula E_CB_ = E_VB_ − E_g_.

**Figure 11:**
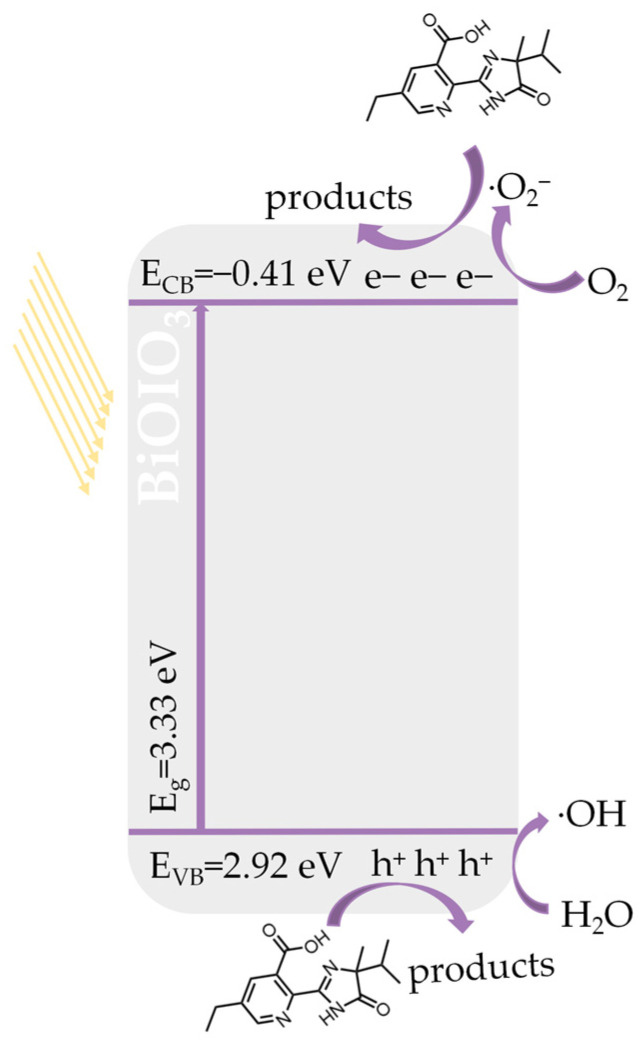
Schematic diagram for photocatalytic elimination mechanism of as-obtained BiOIO_3_ for IMT.

The charge separation and transfer processes of the photocatalytic elimination mechanism of IMT over BiOIO_3_ are illustrated in Figure 11. Upon photoexcitation, e^−^ in BiOIO_3_ are excited and transferred from the valence band (VB) to the conduction band (CB), leaving h^+^ in the valence band. The e^−^ on the CB react with dissolved oxygen in water to form ·O_2_^−^, while the h^+^ remaining in the VB react with water molecules (H_2_O) and OH^−^ to generate ·OH. Among these ROS, e^−^, ·O_2_^−^ and h^+^ act as the primary active species responsible for the photocatalytic elimination of IMT.

### 2.4. Possible Transformation Pathway of IMT and Toxicology Evaluation of Intermediates

**Figure 12:**
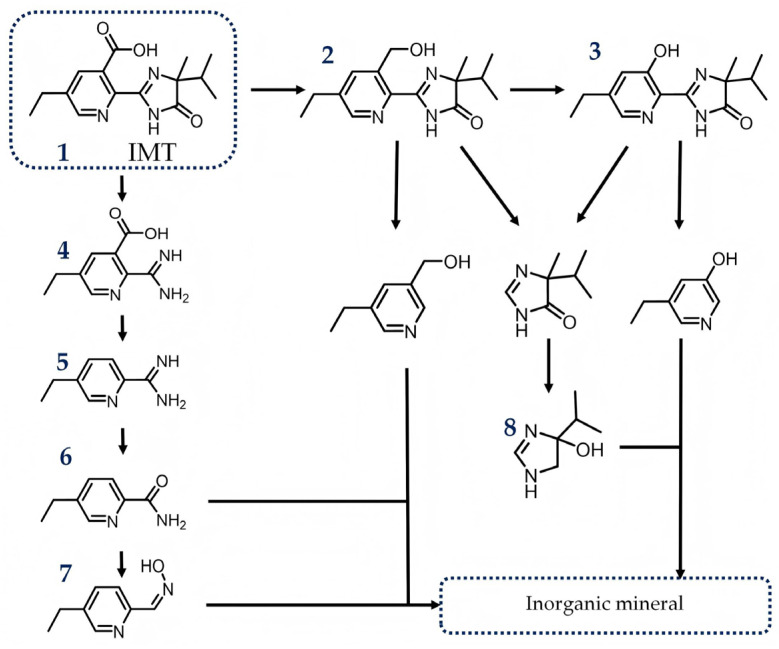
Possible transformation pathway of IMT under Xenon lamp source illumination with as-obtained BiOIO_3_ as photocatalyst.

To elucidate the elimination intermediates and their possible formation pathways of IMT, HPLC-MS/MS in scan mode (ESI positive and negative modes) was used to characterize the intermediates. The mass spectra and the corresponding putative structural formulas for the newly emerged peaks after light source activation are presented in Figure S1. When scanning was performed in ESI positive mode, peaks at *m*/*z* = 276.2, 262.2, 194.1 and 150.2 were observed; in ESI negative mode, peaks at *m*/*z* = 149.0 and 126.9 were detected. The relevant information of the intermediates is summarized in Table S1, and the putative photocatalytic elimination pathways of IMT are proposed as illustrated in Figure 12. Path I: The imidazole ring of IMT is cleaved first, with the subsequent elimination of the carboxylic acid group. Path II: The carboxylic acid group of IMT is initially reacted and then gradually cleaved into small molecules. Ultimately, as shown in Figure S1, the total organic carbon (TOC) test results indicate that as the duration of the photocatalytic reaction increases, IMT eventually decomposes into inorganic mineral.

**Figure 13:**
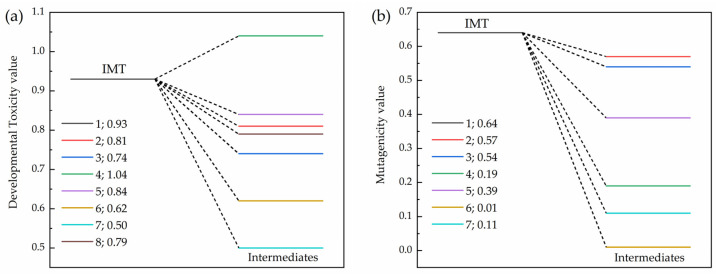
(**a**) Developmental toxicity and (**b**) mutagenicity evaluation of IMT and its main elimination intermediates.

To evaluate the toxicity of the major elimination intermediates of IMT, the toxicity prediction software T.E.S.T (Version 5.1.2) based on the quantitative structure–activity relationship (QSAR) was employed for calculation and assessment, as shown in Figure 13. The developmental toxicity of IMT elimination intermediates 2, 3, 5, 6, 7 and 8 all decreased, while that of intermediate 4 increased to a certain extent. For the mutagenicity prediction, the predicted result of intermediate 8 was negative and thus it was not plotted in Figure 13; the mutagenicity of intermediates 2, 3, 4, 5, 6 and 7 all exhibited a decrease.

### 2.5. Photocatalytic Degradation of 5 Imidazolinone Herbicides Simultaneously

**Figure 14:**
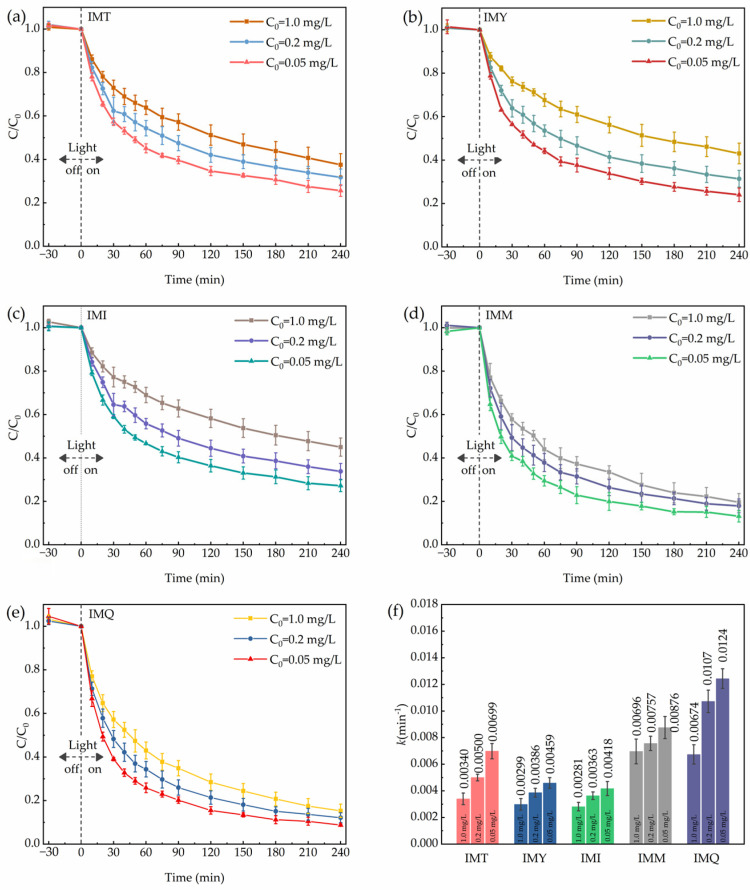
Photocatalytic elimination of IMT (**a**), IMY (**b**), IMI (**c**), IMM (**d**), and IMQ (**e**) by as-obtained BiOIO_3_; (**f**) photocatalytic elimination rat constant (*k*) of as-obtained BiOIO_3_ for five imidazolinone herbicides.

BiOIO_3_ was applied to the simultaneous photocatalytic elimination of five imidazolinone herbicides, including imazethapyr (IMT), imazapyr (IMY), imazapic (IMI), imazamox (IMM) and imazaquin (IMQ), with three initial concentrations set at 1.0 mg/L, 0.2 mg/L and 0.05 mg/L, respectively. The results are presented in Figure 14a–e. BiOIO_3_ exhibited excellent photocatalytic elimination performance for all five imidazolinone herbicides. After 240 min of light irradiation, the elimination efficiencies of BiOIO_3_ for IMT, IMY, IMI, IMM and IMQ all exceeded 55%, with the best photocatalytic degradation effect observed for IMQ (elimination efficiencies is 87.5%). As shown in Figure 14f, the elimination rate constant (*k*) of BiOIO_3_ toward five imidazolinone herbicides reached up to 0.0124 (IMQ, initial concentration: 0.05 mg/L). Furthermore, the lower the initial concentration, the better the elimination performance. This may be attributed to saturated active sites, competitive adsorption of intermediates, and limited generation of reactive oxygen species. These results demonstrate that BiOIO_3_ possesses excellent photocatalytic elimination properties for the five imidazolinone herbicides.

### 2.6. Reusability of BiOIO_3_

**Figure 15:**
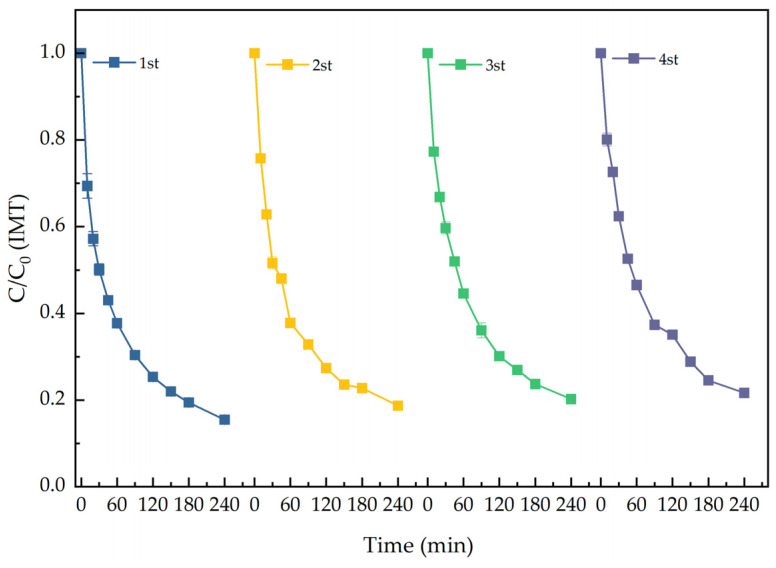
The reusability test of IMT elimination over BiOIO_3_.

The reusability of BiOIO_3_ was evaluated via cyclic photocatalytic elimination experiments. As depicted in Figure 15, BiOIO_3_ retained high elimination efficiency with no obvious decline over four consecutive recycling cycles. The excellent reusability of BiOIO_3_ indicates its promising potential for practical applications.

## 3. Materials and Methods

### 3.1. Reagents, Equipment and Characterizations

Imazapyr (IMY, 99.5%) and imazaquin (IMQ, 98.7%) were purchased from Shanghai Aladdin Biochemical Technology Co., Ltd. (Shanghai, China). Imazapic (IMI, 99.0%) and imazamox (IMM, 99.0%) were procured from Beijing J&K Scientific Technology Co., Ltd. (Beijing, China). Imazethapyr (IMT, 98.0%) was acquired from Shanghai Maclean Biochemical Technology Co., Ltd. (Shanghai, China). All of the following chemical reagents were of analytical grade. Bismuth nitrate pentahydrate (Bi(NO_3_)_3_·5H_2_O), Potassium iodate (KIO_3_), tert-butanol (TBA) and p-benzoquinone (p-BQ) were purchased from Shanghai Energy Chemical Co., Ltd. (Shanghai, China). Potassium bromate (KBrO_3_) and disodium ethylenediaminetetraacetate (EDTA-Na_2_) were acquired from Sinopharm Chemical Reagent Co., Ltd. (Shanghai, China). TiO_2_ (Rutile and Anatase), BiVO_4_, WO_3_ and BiOCl were purchased from Shanghai Aladdin Biochemical Technology Co., Ltd. All chemicals used in this work were commercially available and used without further purification. The working solutions were prepared daily. Doubly distilled water was used in all of the experiments.

The photocatalytic elimination experiment of IMT and five imidazolinone herbicides was conducted via a photocatalytic reaction apparatus (CEL-HXF300, Beijing Zhongjiaojinyuan Technology Co., Ltd., Beijing, China) equipped with a 300 W Xenon lamp. The optical power density was measured by a CEL-NP2000 high-light optical power meter (Beijing Zhongjiaojinyuan Technology Co., Ltd., Beijing, China). All materials are prepared using a WH-Constant temperature drying oven (Tianjin Tester Instrument Co., Ltd., Tianjin, China). The size and morphology of the as-obtained BiOIO_3_ were observed by SEM (ZEISS sigma500, Oberkochen, Germany) and TEM (JEM 2011Plus, Kitaku, Japan). The Brunauer–Emmett–Teller (BET) surface area and pore size analysis was performed by a physical adsorption analyzer (3H-2000PS2) (Bei Shi De, Beijing, China). XRD (Shanghai Aladdin Biochemical Technology Co., Ltd., Shanghai, China) patterns of the samples are acquired over diffraction angles (2θ) of 10–90° by a Bruker (Billerica, MA, USA) D8 ADVANCE X-ray diffractometer with Cu Kα radiation. FTIR spectra were obtained on an IRTracer (Kyoto, Japan) 100 FT-IR Spectrometer with a KBr pellet. XPS data were taken on a Thermo Fisher (Waltham, MA, USA) K-Alpha X-ray photoelectron spectrometer. The UV–Vis DRS data were recorded by a UV3600 UV–Vis spectrophotometer at room temperature with a BaSO_4_ reference standard. The electrochemical measurements (TPC and EIS) were performed in a conventional three-electrode cell on a CHI-760E electrochemical workstation (Shanghai Chenhua Instrument Co., Ltd., Shanghai, China). The PL spectra were measured by an FLS1000/FS5 steady-state and transient fluorescence spectrometer at an excitation wavelength of 420 nm. The TOC test was analyzed by a multi N/C 2100 TOC/TN Analyzer (Analytik Jena, Jena, Germany).

### 3.2. The Preparation Method of BiOIO_3_

Synthesis of BiOIO_3_: A total of 1 mmol of bismuth nitrate pentahydrate (Bi(NO_3_)_3_·5H_2_O) was magnetically stirred in 80 mL of deionized water for 30 min to form a white suspension. Subsequently, 1 mmol of potassium iodate (KIO_3_) was added, and the mixture was stirred continuously for 2 h. The resulting suspension was transferred into a 100 mL Teflon-lined stainless steel autoclave and heated at 160 °C for 12 h in a constant-temperature drying oven. After the reaction, the white solid product was washed with deionized water and anhydrous ethanol, then dried at 70 °C for further use.

### 3.3. Photocatalytic Experiments

In this work, all photocatalytic reactions were performed in a 150 mL reactor fitted with a circulating water system and under Xenon lamp source illumination (the radiation range is 300–2000 nm and the intensity of light ≈ 100 mW/cm^2^). In a typical experiment, 50 mg of photocatalyst was dispersed in 100 mL IMT (initial concentration: 2 mg/L) solution. First, it was stirred for 30 min in dark to establish adsorption–desorption equilibrium between the solution and the catalysts. Then the light source was turned on and the photocatalytic elimination test performed. An aliquot of about 0.80 mL was taken out from the reactor at a given time interval (0, 10, 20, 30, 45, 60, 90, 120, 150, 180 and 240 min) of irradiation and the materials removed for further analysis. In the elimination experiments of 5 imidazolinone herbicides (IMT, IMY, IMI, IMM and IMQ) simultaneously, we investigated the photocatalytic elimination effects at different concentrations (1 mg/L, 0.2 mg/L and 0.05 mg/L). To detect reactive species of the photocatalytic IMT elimination process, several types of scavengers were added into the BiOIO_3_ system. TBA, KBrO_3_, EDTA-Na_2_ and p-BQ were added to the reaction system to capture ·OH, e^−^, h^+^ and ·O_2_^−^ separately. The dosages of all scavengers were 1 mM. In order to detect the produced intermediates of IMT more accurately, 120 mg BiOIO_3_ was dispersed in 100 mL IMT water solution with an initial concentration of 20 mg/L. And the sample intervals were 15, 30, 45, 60, 90, 120, 150, 180, 240, 300, 360, 420, 480, 600 and 720 min.

### 3.4. Analytical Methods

The concentrations of IMT and 5 imidazolinone herbicides were determined by high-performance liquid chromatography (HPLC, Agilent 1200, Santa Clara, CA, USA) and an Agilent 6410B triple-quadrupole mass spectrometer equipped with an electrospray ionization (ESI) interface (Agilent Technologies, Santa Clara, CA, USA), expressed as HPLC-MS/MS. The HPLC was equipped with a CNW Athena C_18_-WP column (3.5 m particle size, 2.1 mm × 50 mm). The triple-quadrupole mass spectrometer was operated in MRM after selected ion monitoring had been performed to detect the concentration of IMT and 5 imidazolinone herbicides. For IMT, the mobile phase was H_2_O-HCOOH 0.1% and acetonitrile (the ratio of H_2_O-HCOOH 0.1% to acetonitrile was 20:80) with a flow rate of 0.30 mL min^−1^. And the quantitative and qualitative ion pairs were 290.2 → 177.2 and 290.2 → 86.1, respectively. In addition, the instrument conditions and MRM data acquisition parameters of HPLC-MS/MS for 5 imidazolinone herbicides are shown in Table S2. The mobile phase was H_2_O-HCOOH 0.1% and acetonitrile (the ratio of H_2_O-HCOOH 0.1% to acetonitrile was 60:40) with a flow rate of 0.30 mL min^−1^.

### 3.5. Toxicity Evaluation

T.E.S.T (Toxicity Estimation Software Tool) (version 5.1.2) is a quantitative structure–activity relationship (QSAR) software for toxicity prediction developed by the United States Environmental Protection Agency (US EPA). Inputting the chemical structure of IMT and the intermediate products into the software T.E.S.T, the predicted developmental toxicity and mutagenicity values are then calculated.

## 4. Conclusions

This study successfully developed a high-performance BiOIO_3_ photocatalyst via a simple hydrothermal route. Comprehensive characterizations confirmed its well-defined 2D lamellar morphology, pure crystal structure, and favorable band alignment. The unique internal polarization electric field of BiOIO_3_ significantly enhances charge separation, endowing it with superior photocatalytic activity for IMT elimination compared to conventional TiO_2_ and WO_3_. Mechanistic investigations clarified the dominant role of e^−^, h^+^, and ·O_2_^−^ in the degradation process, and the proposed transformation pathways involve cleavage of the imidazole ring and carboxylic acid group, leading to non-toxic inorganic products. Toxicity evaluation verified reduced environmental risks of key intermediates. Moreover, BiOIO_3_ exhibited excellent performance in the simultaneous elimination of five imidazolinone herbicides, especially at low initial concentrations. On the basis of the above results, BiOIO_3_ is proven to possess extensive application prospects for the efficient photocatalytic elimination of imidazolinone herbicides.

## Data Availability

The data presented in this study are available upon request from the corresponding author.

## Supplementary Materials

The following supporting information can be downloaded at: https://www.mdpi.com/article/10.3390/molecules31081361/s1, Figure S1: The mass spectrum of IMT elimination intermediate products; Figure S2: The total organic carbon (TOC) removal efficiency of BiOIO_3_; Table S1: The essential information of IMT and its elimination intermediates; Table S2: MRM transitions and other HPLC-MS/MS parameters of investigated 5 imidazolinone herbicides.
