# Supplementary material for: Efficient Photocatalytic Elimination of Imidazolinone Herbicides by Bismuth-Based Photocatalyst BiOIO3
Source: Molecules. 2026 Apr 21;31(8):1361. doi: 10.3390/molecules31081361 (PMC13118289; doi:10.3390/molecules31081361)
Supplement: Supplementary file 1 [file molecules-31-01361-s001.zip › molecules-4238232-supplementary.pdf]

Supplementary Materials

Efficient Efficient Photocatalytic Elimination of Imidazolinone Herbicides by Bismuth-Based Photocatalyst BiOIO3

Weili Yu [1], Yan Tian [2], Mengyu Guo [1], Shuping Tong [1], Chengshuai Li [1], Bingjie Zhang [1],* and Yongqiang Ma [3]

1 Shandong Key Laboratory for Green Prevention and Control of Agricultural Pests, Shandong Academy of Pesticide Sciences, Jinan 250033, China; 313304200@163.com (W.Y.); mengyuguo566@163.com (M.G.); 13989046652@163.com (S.T.); liucling2004@163.com (C.L.).

2 Guangxi Zhuang Autonomous Region Ecological Environment Monitoring Center, Nanning 530012, China; ttyezi96@163.com (Y.T.).

3 Department of Applied Chemistry, College of Science, China Agricultural University, Beijing 100193, China; mayongqiang@cau.edu.cn (Y.M.).

* Correspondence: zhangbj0923@126.com (B.Z.)

The formulas

$$\ln(C_0/C) = kt$$

where C is the residual concentration at reaction time t, C_0 is the initial concentration, k is the pseudo-first-order rate constant.

$$(\alpha hv)^{1/2} = A\ (hv - E_g)$$

where α is the absorption coefficient, h is the Planck constant, v is the frequency of vibration, A is a proportional constant, E_g is the bandgap.

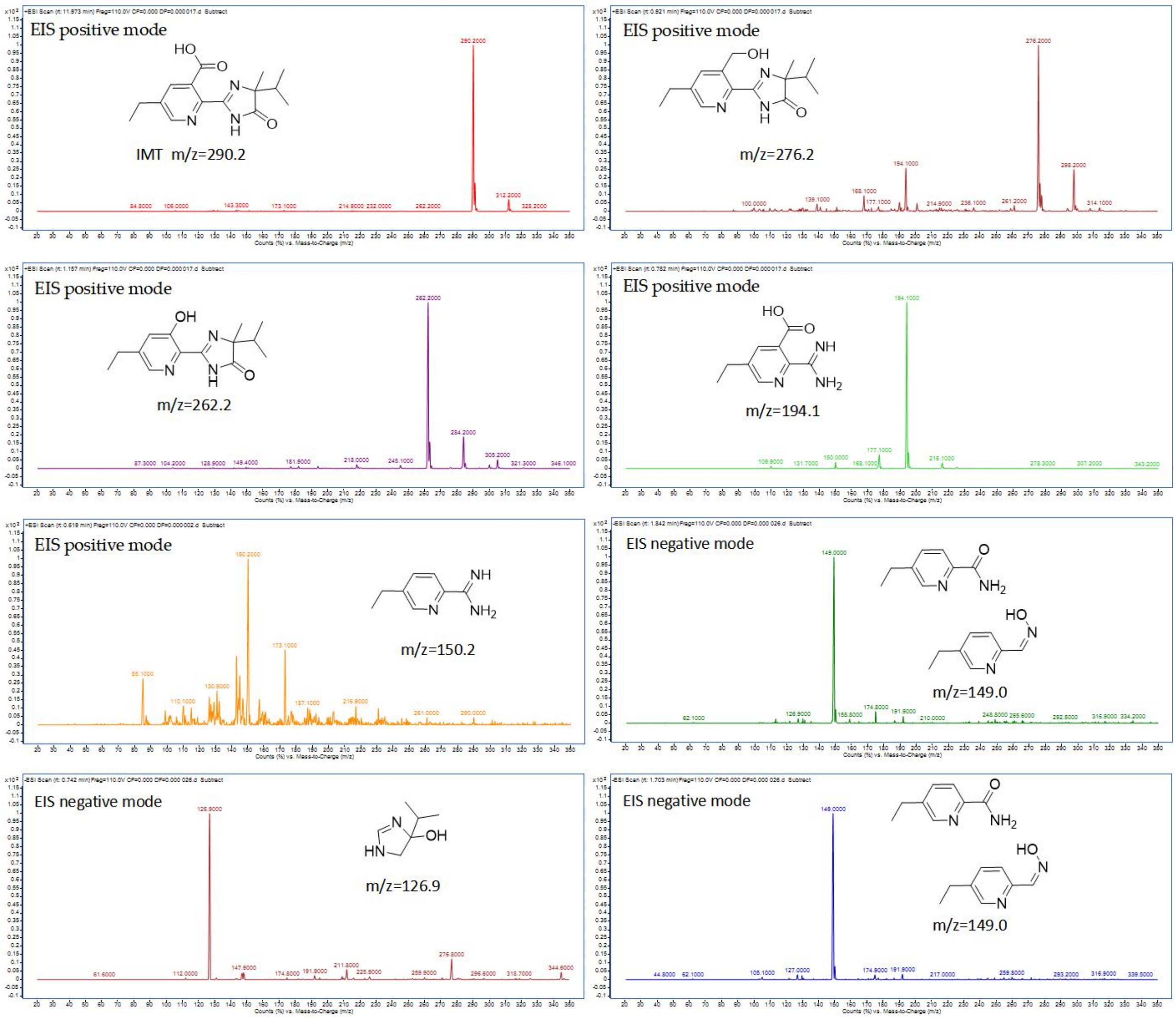

Figure S1. The mass spectrum of IMT elimination intermediate products.

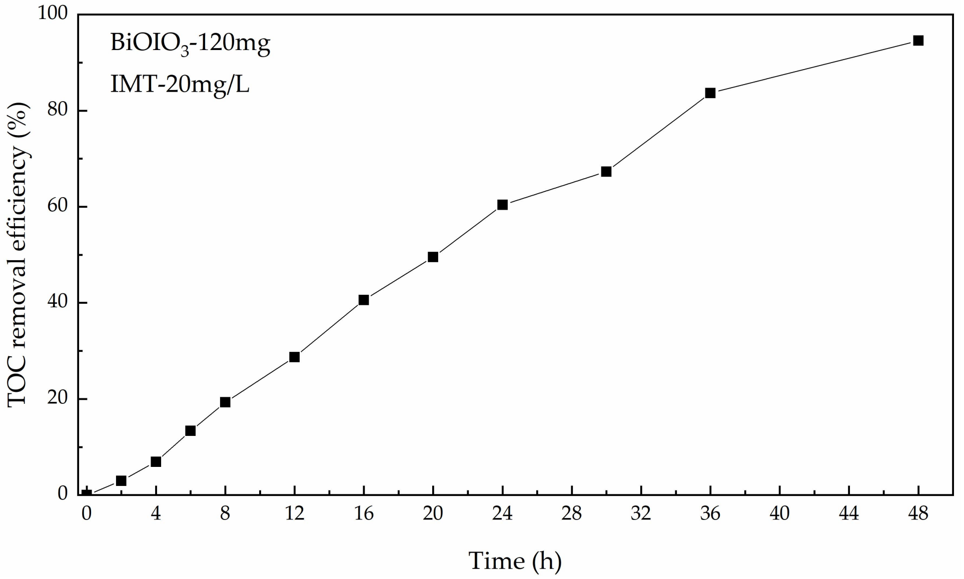

Figure S2. The total organic carbon (TOC) removal efficiency of $BiOIO_3$.

Table S1. The essential information of IMT and its elimination intermediates.

No.	Molecular formulas	CAS number	Molecular formula	Structure formulas
1	289.33	81335-77-5	$C_{15}H_{19}N_3O_3$	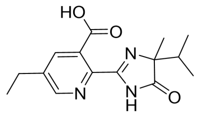
2	275.35	/	$C_{15}H_{21}N_3O_2$	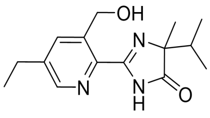
3	261.33	/	$C_{14}H_{19}N_3O_2$	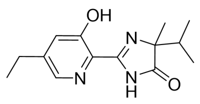
4	193.21	1312104-20-3	$C_9H_{11}N_3O_2$	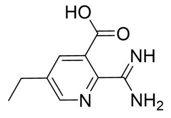
5	149.19	1179533-38-0	$C_8H_{11}N_3$	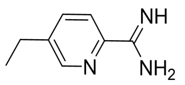
6	150.18	13509-17-6	$C_8H_{10}N_2O$	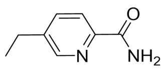
7	150.18	10177-28-3	$C_8H_{10}N_2O$	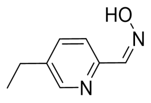
8	128.18	/	$C_6H_{12}N_2O$	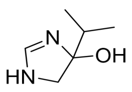

Table S2. MRM transitions and other HPLC-MS/MS parameters of investigated 5 imidazolinone herbicides.

No.	Pesticide	Molecular structure	Prec Ion	Prod Ion	Frag (V)	CE
1	IMT	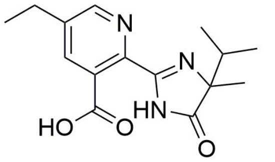	262.0	234.0	120	15
				217.0*	110	15
2	IMY	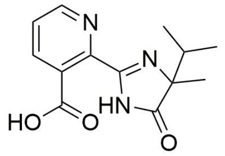	276.1	248.0	120	15
				231.0*	120	20
3	IMI	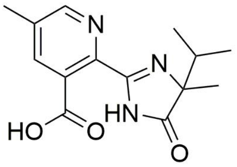	290.2	177.2*	135	30
				86.1	135	30
4	IMM	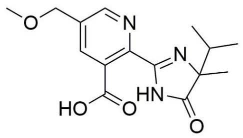	306.0	278.1	135	15
				261.0*	120	20
5	IMQ	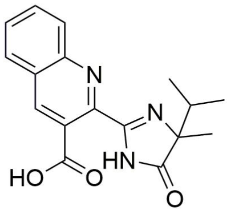	312.1	267.0	135	20
				198.9*	135	30

*is the quantitative ion.
